# Preoperative CT-based deep learning radiomics model to predict lymph node metastasis and patient prognosis in bladder cancer: a two-center study

**DOI:** 10.1186/s13244-023-01569-5

**Published:** 2024-01-25

**Authors:** Rui Sun, Meng Zhang, Lei Yang, Shifeng Yang, Na Li, Yonghua Huang, Hongzheng Song, Bo Wang, Chencui Huang, Feng Hou, Hexiang Wang

**Affiliations:** 1 Department of Radiology, The Affiliated Hospital of Qingdao University, Qingdao, 266003 Shandong China; 2 Department of Radiology, Qingdao Center Hospital, Qingdao, 266042 Shandong China; 3 Department of Radiology, Shandong Provincial Hospital Affiliated to Shandong First Medical University, Jinan, 250000 Shandong China; 4 Department of Radiology, The People’s Hospital of Zhangqiu Area, Jinan, 250200 Shandong China; 5 Department of Radiology, The Puyang Oilfield General Hospital, Puyang, 457001 Henan China; 6 Department of Research Collaboration, R&D Center, Beijing Deepwise & League of PHD Technology Co., Ltd., Beijing, 100080 China; 7 Department of Pathology, The Affiliated Hospital of Qingdao University, Qingdao, 266003 Shandong China

**Keywords:** Deep learning, Nomogram, Urinary bladder neoplasms, Lymphatic metastasis, Computed tomography

## Abstract

**Objective:**

To establish a model for predicting lymph node metastasis in bladder cancer (BCa) patients.

**Methods:**

We retroactively enrolled 239 patients who underwent three-phase CT and resection for BCa in two centers (training set, *n* = 185; external test set, *n* = 54). We reviewed the clinical characteristics and CT features to identify significant predictors to construct a clinical model. We extracted the hand-crafted radiomics features and deep learning features of the lesions. We used the Minimum Redundancy Maximum Relevance algorithm and the least absolute shrinkage and selection operator logistic regression algorithm to screen features. We used nine classifiers to establish the radiomics machine learning signatures. To compensate for the uneven distribution of the data, we used the synthetic minority over-sampling technique to retrain each machine-learning classifier. We constructed the combined model using the top-performing radiomics signature and clinical model, and finally presented as a nomogram. We evaluated the combined model’s performance using the area under the receiver operating characteristic, accuracy, calibration curves, and decision curve analysis. We used the Kaplan–Meier survival curve to analyze the prognosis of BCa patients.

**Results:**

The combined model incorporating radiomics signature and clinical model achieved an area under the receiver operating characteristic of 0.834 (95% CI: 0.659–1.000) for the external test set. The calibration curves and decision curve analysis demonstrated exceptional calibration and promising clinical use. The combined model showed good risk stratification performance for progression-free survival.

**Conclusion:**

The proposed CT-based combined model is effective and reliable for predicting lymph node status of BCa patients preoperatively.

**Critical relevance statement:**

Bladder cancer is a type of urogenital cancer that has a high morbidity and mortality rate. Lymph node metastasis is an independent risk factor for death in bladder cancer patients. This study aimed to investigate the performance of a deep learning radiomics model for preoperatively predicting lymph node metastasis in bladder cancer patients.

**Key points:**

• Conventional imaging is not sufficiently accurate to determine lymph node status.

• Deep learning radiomics model accurately predicted bladder cancer lymph node metastasis.

• The proposed method showed satisfactory patient risk stratification for progression-free survival.

**Graphical Abstract:**

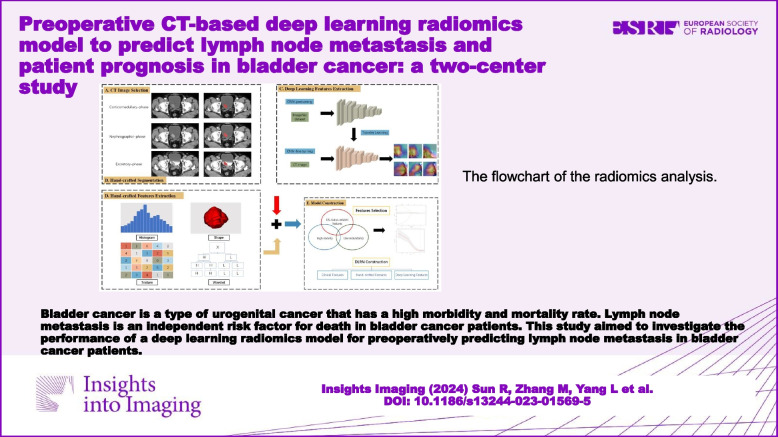

**Supplementary Information:**

The online version contains supplementary material available at 10.1186/s13244-023-01569-5.

## Background

Bladder cancer (BCa) is a type of urogenital cancer that has a high morbidity and mortality rate [1]. The most common metastatic sites are lymph nodes (LN) [2]. Lymph node metastasis (LNM) affects the survival rate of BCa patients. For BCa patients with LNM, the 5-year cancer-specific survival rate was 27.7% [3], which is observably lower than that of patients without LNM. As a result, accurate preoperative prediction of LNM is of significance for disease staging, therapy selection, and survival prediction [4]. Examinations such as ultrasonography, CT, and MRI are all commonly used to diagnose BCa, however, their efficacy in identifying metastatic malignant LN is unsatisfactory [5]. Needle biopsy is an effective but invasive method; furthermore, the sample selection of the nodes can sometimes cause false negatives [6]. Because of the limitations of current diagnostic methods, there is an essential requirement for a non-invasive and precise method to predict LNM with BCa.

Three-phase CT has become an important clinical examination tool because of its economical and rapid imaging. Grobmyer et al. [7] evaluated LNM based on the size of retroperitoneal and pelvic LN captured in CT images, obtaining a diagnostic sensitivity of only 40%, thereby failing to meet the requirements of clinical precision diagnosis and treatment.

Heterogeneity exists within tumors and is expressed on various spatial scales, including the genetic, cellular, molecular, and radiological level [8]. Thus, we often need to obtain a portion of the tumor tissue by invasive methods. Radiomics — the high-throughput capture and analysis of a vast amount of advanced quantitative imaging features from digital medical images — is a potential non-invasive approach to analyze the entire tumor [9]. While radiomics can quantify heterogeneity within tumors, partial volume effects may lead to inaccurate quantification of heterogeneity in small lesions. Hatt et al. [10] demonstrated that radiomics texture features achieved relatively poor prognostic accuracy for tumors < 10 cm^3^. Both positive and negative LN tumors are small, so radiomics may not be appropriate for LN analysis. Thus, the objective of our research was to develop a LNM prediction model based on three-phase CT images of primary lesions.

Deep learning (DL) has been widely used in medical imaging as the most effective method for learning feature expressions [11]. This technique can enable more accurate correlation between radiomics models and disease characteristic prediction. Nevertheless, the use of CT-based hand-crafted radiomics (HCR) features and DL features to predict the LNM of BCa has not yet been studied.

We aim to build a deep learning radiomics model based on three-phase CT using a two-center dataset to accurately predict the LNM status for BCa patients preoperatively.

## Methods

### Patient selection

This retrospective research was approved by the hospital’s review board, waiving the requirement for patient informed consent. We selected BCa patients who were treated between March 2008 and June 2022 in accordance with our inclusion and exclusion criteria. Patients were subject to the following inclusion criteria: (a) pathologically confirmed urothelial carcinoma; (b) standard pelvic three-phase CT performed < 20 days before surgery; (c) extended pelvic lymph node dissection (up to the aortic bifurcation); (d) adequate follow-up examinations. The following were the exclusion criteria: (a) patients with other tumor disease simultaneously; (b) incomplete clinical or imaging data; (c) patients who were given preoperative care, such as chemotherapy, radiotherapy, or immunotherapy. Ultimately, we included 239 patients in our research: we used data collected from 185 patients of the Affiliated Hospital of Qingdao University as the training set, and data collected from 54 patients of Shandong Provincial Hospital Affiliated to Shandong First Medical University as the external test set.

We gathered the following clinical information from medical records and CT images: patient age, patient gender, tumor location, shape (cauliflower-like, papillary, or mound-like lesions), size, calcification (yes or no), cystic necrosis (yes or no), boundary (clear or unclear), number (solitary or multiple), stalk (absent or present), CT reported T stage, CT reported LN status, and CT value of lesions in the three phases (corticomedullary-phase, nephrographic-phase, and excretory-phase). Patients with observable abdominal LN > 10 mm or pelvic LN > 8 mm in the maximal short-axis diameter were considered as clinically LN-positive [12]. Two experienced radiologists reviewed the CT images and resolved any discrepant interpretations through discussion.

### CT image acquisition

All patients underwent three-phase CT examination. Supplementary Table S1 displays the CT acquisition settings. The three-phase (corticomedullary-phase, nephrographic-phase, and excretory-phase) images were acquired at 25 s, 75 s, and 300 s after the bolus-triggering threshold of 120 HU had been reached at the thoracoabdominal aorta junction, respectively.

### Lesion segmentation and feature extraction

**Fig. 1:**
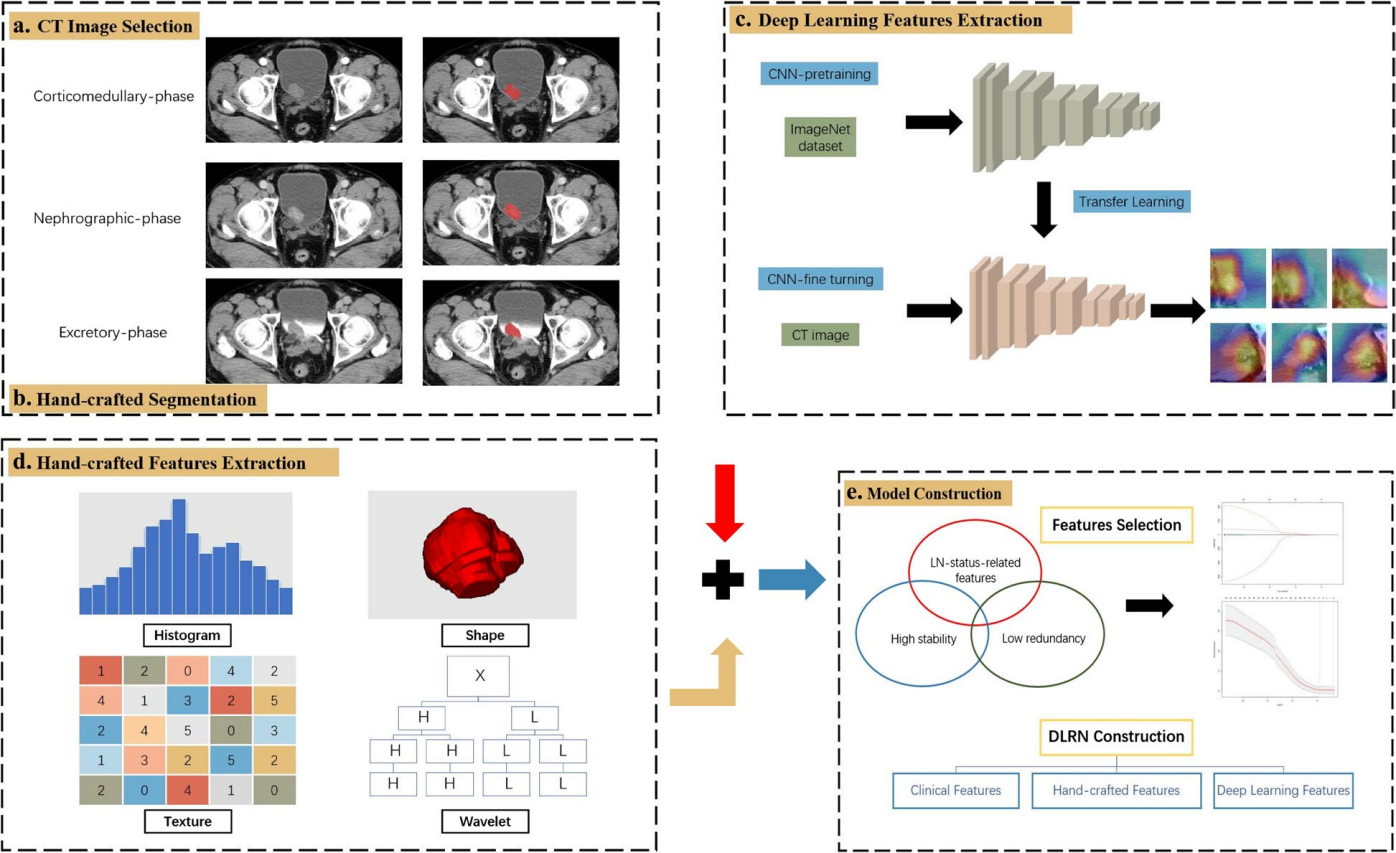
Flowchart of the radiomics analysis

The flow of radiomics is shown in Fig. 1. A radiologist manually performed the region of interest (ROI) segmentation for all tumor lesions using the ITK-SNAP software (version 3.8.0, http://www.itksnap.org) [13]. The maximum extent of the ROI was outlined layer by layer along the edge of the lesion, avoiding vesical stones and ureters. The 3D-ROI is then automatically generated by the software. After a month, the same radiologist randomly selected 94 patients for a second ROI manual segmentation to assess intraobserver reliability. Another radiologist performed ROI manual segmentation in the same way for the interobserver agreement assessment. Radiomics features with intra-/inter-observer correlation coefficients (ICCs) > 0.8 were included in the follow-up study.

We used the deep convolution network ResNet18 to extract the DL features with the stochastic gradient descent optimizer for training [14]. We pre-trained the model on the Onekey platform for transfer learning.

We processed the radiomics features using the combat compensation methodology to eliminate the influence of different protocols and CT scanners in the two centers, while retaining texture pattern characteristics [15].

### Radiomics signature development

We unified all radiomics features according to z-score. We then dimensionally reduced the features before building the model. First, we used the Minimum Redundancy Maximum Relevance (mRMR) algorithm to retain 50 features with high correlation and low redundancy. Then, we used the least absolute shrinkage and selection operator (LASSO) logistic regression algorithm to reduce the dimensionality and retain 12 features showing the best predictive potential. Finally, we used nine machine-learning classifiers to develop machine-learning signatures: support vector machine (SVM), logistic regression (LR), Extreme Gradient Boosting (XGBoost), NaiveBayes, Adaptive Boosting (AdaBoost), Light Gradient Boosting Machine (LightGBM), k-nearest neighbor (KNN), Multilayer Perceptron (MLP), and GradientBoosting. Initially, we trained each machine-learning signature without subsampling, then retrained them using the synthetic minority oversampling technique (SMOTE) [16].

### Combined model construction

We applied univariate logistic regression to screen clinical characteristics and CT features related to LNM of BCa. Then, we input the features with *p* < 0.05 into the multivariate logistic regression to create the clinical model. We used backward stepwise selection with a likelihood ratio test and Akaike’s information criterion as the stopping rule. We created the combined model by incorporating the risk factors of the clinical model and the radiomics signature achieving the best predictive performance. The combined model presented as a radiomics nomogram. We used the area under the receiver operating characteristic curve (AUC) and accuracy to judge the performance of the clinical model, radiomics signatures, and combined model. We used calibration curves to test the models' fitting and decision curve analysis (DCA) to judge the clinical dependability and practicability of the models.

### Clinical endpoints and follow-up surveillance

For the first two years after surgery, patients were subject to routine imaging methods every 3–6 months, and then annually. The observed index was progression-free survival (PFS). PFS refers to the time between surgery and the survival endpoint, such as detection of tumor recurrence in imaging data, lesion metastasis, the date of the last follow-up, or death. The deadline for follow-up was June 30, 2022. We used Kaplan–Meier survival curves to analyze the prognosis and the log-rank test to assess differences in survival curves.

### Statistical analysis

We used SPSS 26.0 software, R software (version 4.2.2, www.r-project.org), and Python (version3.9.7, www.python.org) for statistical analysis. We compared categorical variables using the chi-square test or Fisher’s exact test. We analyzed continuous variables using the independent sample *t*-test or the Mann–Whitney *U* test. We regarded *p* < 0.05 as statistically significant.

## Results

### Clinical feature selection and clinical model construction

Table 1 details the clinical information and CT features of the patients with BCa in the training and test sets. The results of univariate and multivariate logistic regression were shown in Table 2. Univariate logistic regression analysis revealed that three clinical characteristics substantially contributed to the prediction of LN status in BCa patients: stalk presence, CT reported T stage, and CT reported LN status (each having *p* < 0.05). According to the results of the multivariate logistic regression, stalk presence and CT-reported LN status were independent predictors of LNM in BCa. The clinical model's AUC values were 0.764 (95% CI: 0.697–0.831) in the training set and 0.624 (95% CI: 0.402–0.846) in the external test set.

**Table 1:** Baseline information of the patients

		Training set (*n* = 185)	External test set (*n* = 54)	*p*
Age (years)		68.04 ± 10.00	64.37 ± 12.53	0.052
Gender	Male	155	48	0.356
	Female	30	6	
Location	Bladder triangle	52	12	0.390
	Other part of the bladder	133	42	
Shape	Cauliflower-like	95	31	< 0.001
	Papillary	31	19	
	Mound-like	59	4	
Size		4.44 ± 2.17	2.58 ± 1.12	< 0.001
Calcification	No	156	46	0.878
	Yes	29	8	
Cystic necrosis	No	153	49	0.151
	Yes	32	5	
Tumor boundary	Clear	115	49	< 0.001
	Unclear	70	5	
Number	Solitary	149	43	0.882
	Multiple	36	11	
Stalk	absent	148	27	< 0.001
	present	37	27	
CT reported T stage	Ta-T2	138	45	0.182
	T3-T4	47	9	
CT reported LN status	N0	161	47	0.998
	N1-3	24	7	
CT value in corticomedullary phase		64.17 ± 24.87	75.87 ± 21.52	0.002
CT value in nephrographic phase		75.86 ± 20.93	81.52 ± 17.11	0.071
CT value in excretory phase		74.75 ± 17.98	90.35 ± 50.67	0.030

**Table 2:** Logistic regression analysis of the risk factors for LNM

	Univariate logistic analysis		Multivariate logistic analysis	
	OR (95%CI)	*p*	OR (95%CI)	*p*
Age	1.000 (0.965–1.037)	0.992		
Gender	1.217 (0.479–3.095)	0.679		
Location	1.037 (0.909–1.184)	0.588		
Shape	1.104 (0.742–1.642)	0.625		
Size	0.954 (0.807–1.128)	0.584		
Calcification	0.777 (0.275–2.191)	0.633		
Cystic necrosis	1.367 (0.559–3.342)	0.494		
Tumor boundary	1.253 (0.606–2.589)	0.543		
Number	0.597 (0.303–1.177)	0.137		
Stalk	0.178 (0.041–0.776)	0.022	0.160 (0.032–0.802)	0.026
CT reported T stage	3.662 (1.721–7.794)	0.001	2.009 (0.833–4.848)	0.120
CT reported LN status	16.190 (6.001–43.683)	0	17.049 (5.986–48.558)	0
CT value in corticomedullary phase	0.990 (0.974–1.006)	0.203		
CT value in nephrographic phase	0.999 (0.982–1.017)	0.935		
CT value in excretory phase	1.000 (0.980–1.020)	0.995		

*OR* odds ratio, *CI* confidence interval

### Radiomics signature building and testing

**Fig. 2:**
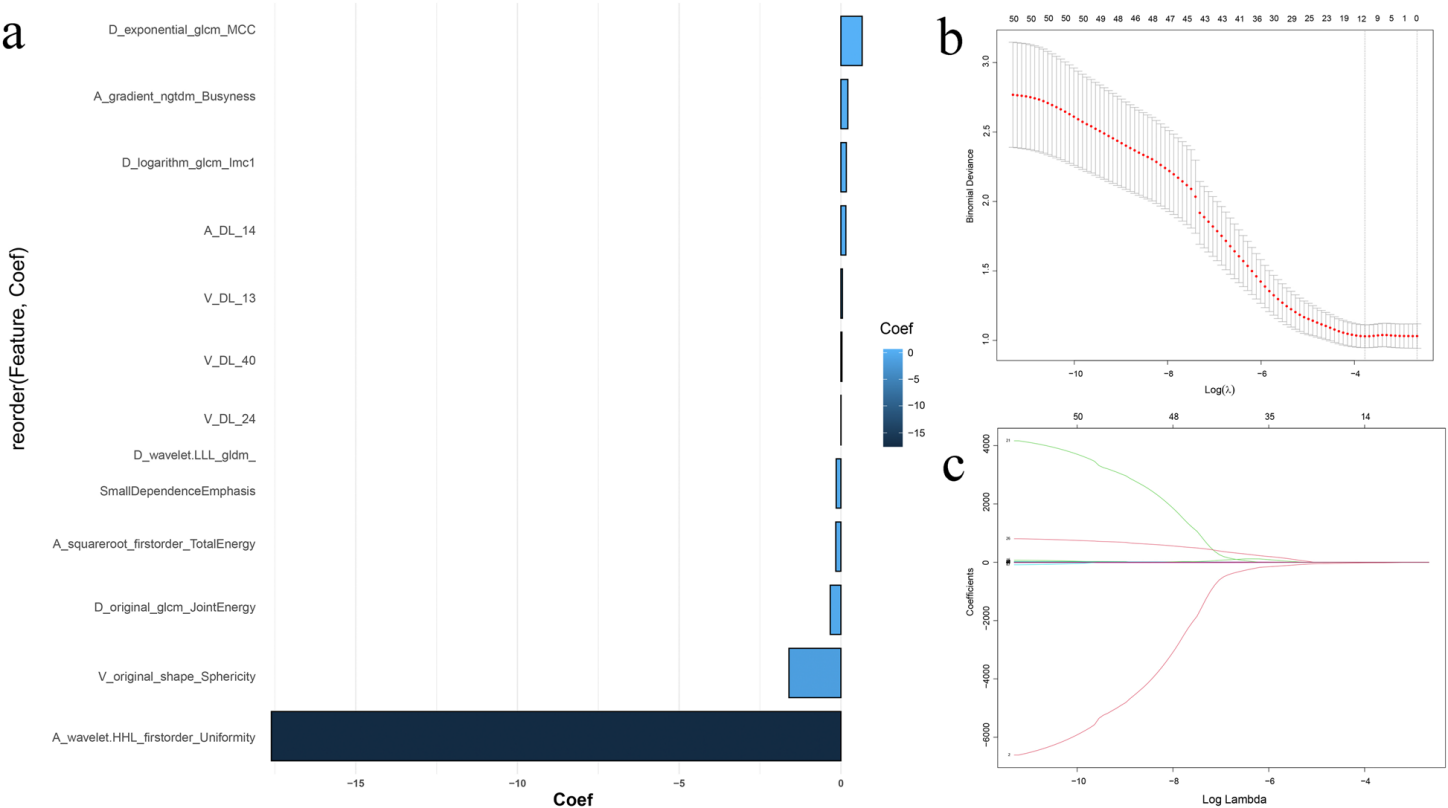
Features were selected by LASSO regression model. **a** The 12 features with non-zero coefficients and the roles of each feature that contributed to the model are shown. **b** Tuning parameter (λ) selection in the LASSO model. **c** The coefficients have been plotted vs. log(λ)

In total, 2645 HCR features were extracted from each ROI (ICCs > 0.8). We combined 2645 HCR features and 384 DL features and included them in subsequent experiments. We used mRMR to retain 50 features, then we used LASSO to reduce the dimension of the dataset and select eight HCR features and four DL features to build the subsequent radiomics signatures (Fig. 2). Tables 3 and 4 show the forecasting ability of all radiomics machine learning signatures without and with SMOTE. For the external test set, the NaiveBayes classifier trained with the combined HCR-DL features achieved the highest AUC of 0.764 (95% CI: 0.604–0.924) without using the SMOTE algorithm. Using the SMOTE algorithm, the LightGBM classifier trained with the combined HCR-DL features achieves the best performance, with an AUC of 0.893 (95% CI: 0.769–1.000) on the external test set. Overall, the SMOTE algorithm improved the AUC result of five radiomics signatures: SVM, LR, XGBoost, LightGBM, and GradientBoosting; and slightly reduced the AUC of four radiomics signatures: NaiveBayes, AdaBoost, KNN, and MLP.

**Table 3:** The predictive performance results of radiomics machine learning signatures without SMOTE

Set	Classifier	AUC (95%CI)	ACC	SEN	SPE	PPV	NPV
Training	SVM	0.932 (0.889–0.975)	0.795	0	1.000	0	0.795
	LR	0.749 (0.660–0.839)	0.811	0.105	0.993	0.800	0.811
	XGBoost	1 (1.000–1.000)	0.995	0.974	1.000	1.000	0.993
	NaiveBayes	0.720 (0.631–0.809)	0.373	1.000	0.211	0.247	1
	AdaBoost	0.879 (0.828–0.930)	0.854	0.447	0.959	0.739	0.870
	LightGBM	0.932 (0.888–0.977)	0.805	0.053	1.000	1.000	0.803
	KNN	0.781 (0.715–0.848)	0.805	0.237	0.952	0.563	0.828
	MLP	0.745 (0.657–0.833)	0.795	0	1.000	0	0.795
	GradientBoosting	0.945 (0.895–0.996)	0.843	0.237	1.000	1.000	0.835
External test	SVM	0.659 (0.465–0.853)	0.815	0	1.000	0	0.815
	LR	0.689 (0.480–0.897)	0.815	0	1.000	0	0.815
	XGBoost	0.720 (0.524–0.917)	0.815	0	1.000	0	0.815
	NaiveBayes	0.764 (0.604–0.924)	0.704	0.700	0.705	0.350	0.912
	AdaBoost	0.699 (0.510–0.888)	0.815	0.100	0.977	0.500	0.827
	LightGBM	0.639 (0.447–0.830)	0.815	0	1.000	0	0.815
	KNN	0.682 (0.531–0.833)	0.796	0	0.977	0	0.811
	MLP	0.727 (0.570–0.884)	0.815	0	1.000	0	0.815
	GradientBoosting	0.616 (0.385–0.847)	0.815	0	1.000	0	0.815

*AUC* area under the receiver operating characteristic curve, *CI* confidence interval, *ACC* accuracy, SEN sensitivity, *SPE* specificity, *PPV* positive predictive value, *NPV* negative predictive value

**Table 4:** The predictive performance results of radiomics machine learning signatures with SMOTE

Set	Classifier	AUC (95%CI)	ACC	SEN	SPE	PPV	NPV
Training	SVM	0.908 (0.873–0.943)	0.847	0.925	0.769	0.800	0.911
	LR	0.765 (0.711–0.819)	0.714	0.748	0.680	0.700	0.730
	XGBoost	1 (1.000–1.000)	0.997	1.000	0.993	0.993	1.000
	NaiveBayes	0.815 (0.767–0.863)	0.616	0.993	0.238	0.566	0.972
	AdaBoost	0.887 (0.850–0.924)	0.793	0.871	0.714	0.753	0.847
	LightGBM	0.969 (0.951–0.987)	0.915	0.918	0.912	0.912	0.918
	KNN	0.959 (0.941–0.977)	0.820	0.980	0.660	0.742	0.970
	MLP	0.827 (0.780–0.874)	0.718	0.810	0.626	0.684	0.767
	GradientBoosting	0.962 (0.943–0.981)	0.864	0.891	0.837	0.845	0.885
External test	SVM	0.686 (0.486–0.887)	0.833	0.500	0.909	0.556	0.889
	LR	0.727 (0.529–0.925)	0.759	0	0.932	0	0.804
	XGBoost	0.782 (0.641–0.922)	0.815	0.200	0.955	0.500	0.840
	NaiveBayes	0.759 (0.596–0.923)	0.741	0.600	0.773	0.375	0.895
	AdaBoost	0.676 (0.456–0.896)	0.759	0.400	0.841	0.364	0.860
	LightGBM	0.893 (0.769–1.000)	0.852	0.400	0.955	0.667	0.875
	KNN	0.673 (0.523–0.823)	0.593	0.500	0.614	0.227	0.844
	MLP	0.727 (0.575–0.880)	0.759	0.300	0.864	0.333	0.844
	GradientBoosting	0.807 (0.631–0.982)	0.815	0.500	0.886	0.500	0.886

*AUC* area under the receiver operating characteristic curve, *CI* confidence interval, *ACC* accuracy, *SEN* sensitivity, *SPE* specificity, *PPV* positive predictive value, *NPV* negative predictive value

### Construction and effectiveness of the combined model

**Fig. 3:**
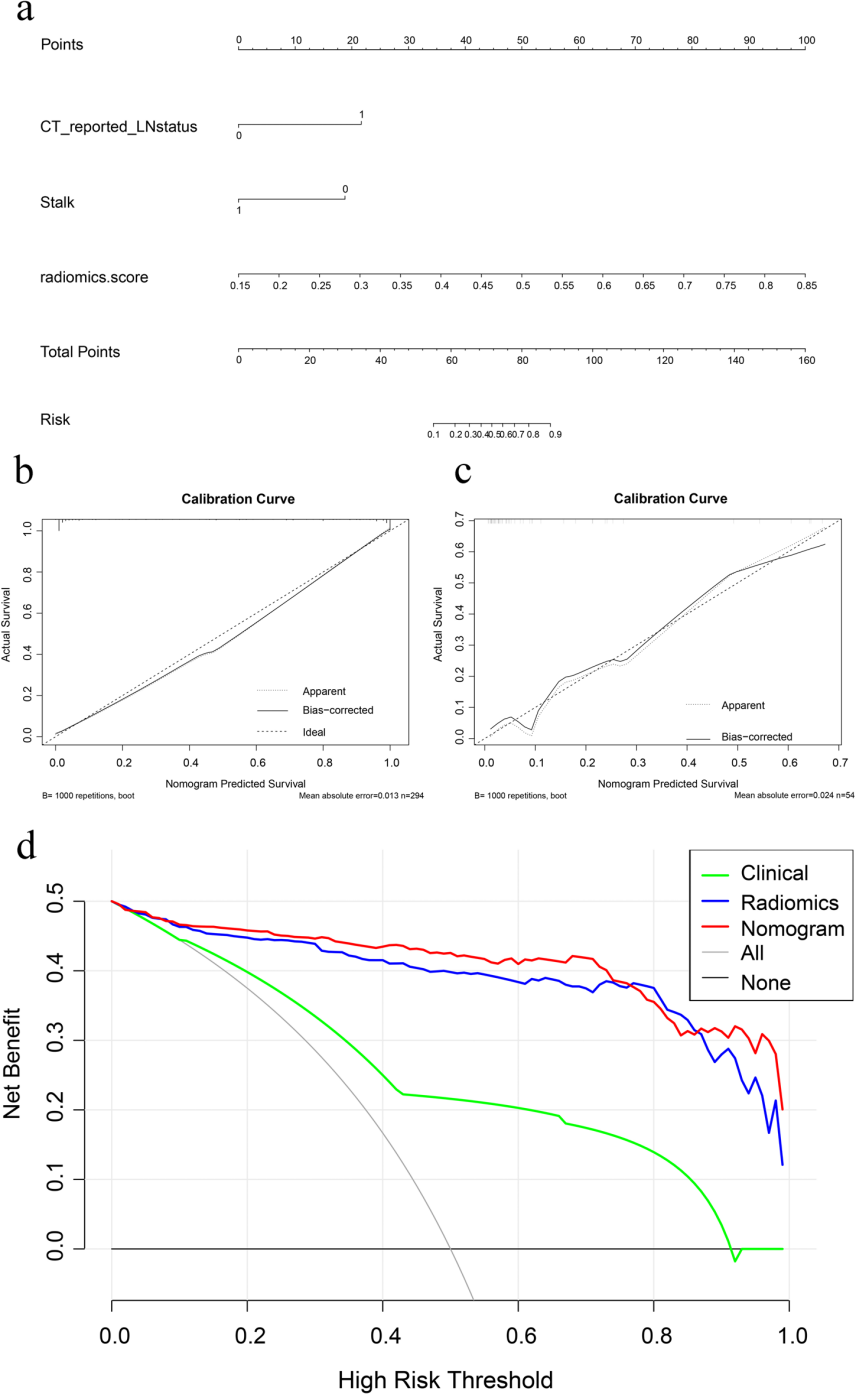
(**a**) Nomogram (**b**, **c**) Calibration curve of the nomogram in the training set and external test set, respectively. **d** DCA for the nomogram

To offer doctors a convenient tool for preoperative prediction of LNM in BCa patients, the combined model was presented as a nomogram. We constructed the combined model by integrating the top-performing radiomics signature (LightGBM-SMOTE) and the clinical factor of significant importance (Fig. 3A). Table 5 shows the predictive performance of the combined model for the training set and external test set. For the external test set, the AUC of combined model (AUC: 0.834, 95% CI: 0.659–1.000) was lower than that of the radiomics signature (AUC: 0.893, 95% CI: 0.769–1.000). However, combined model achieved a higher prediction accuracy (0.870) than the radiomics signature (0.852) on the external test set. Figure 3B and C display the calibration curves of combined model, revealing that it was suitable for both sets. The DCA demonstrated that combined model provided better clinical utility than the radiomics signature (Fig. 3D). Therefore, the combined model achieves the best clinically applicable performance.

**Table 5:** Results of combined model, radiomics signature, and the clinical model predictive ability for LNM status

Set	Model	AUC (95%CI)	ACC	SEN	SPE	PPV	NPV
Training	Combined model	0.980 (0.967–0.993)	0.932	0.939	0.925	0.926	0.938
	Radiomics signature	0.969 (0.951–0.987)	0.915	0.918	0.912	0.912	0.918
	Clinical model	0.764 (0.697–0.831)	0.843	0.395	0.959	0.714	0.860
External test	Combined model	0.834 (0.659–1.000)	0.870	0.400	0.977	0.800	0.878
	Radiomics signature	0.893 (0.769–1.000)	0.852	0.400	0.955	0.667	0.875
	Clinical model	0.624 (0.402–0.846)	0.833	0.300	0.955	0.600	0.857

*AUC* area under the receiver operating characteristic curve, *CI* confidence interval, *ACC* accuracy, *SEN* sensitivity, *SPE* specificity, *PPV* positive predictive value, *NPV* negative predictive value

### Risk stratification

**Fig. 4:**
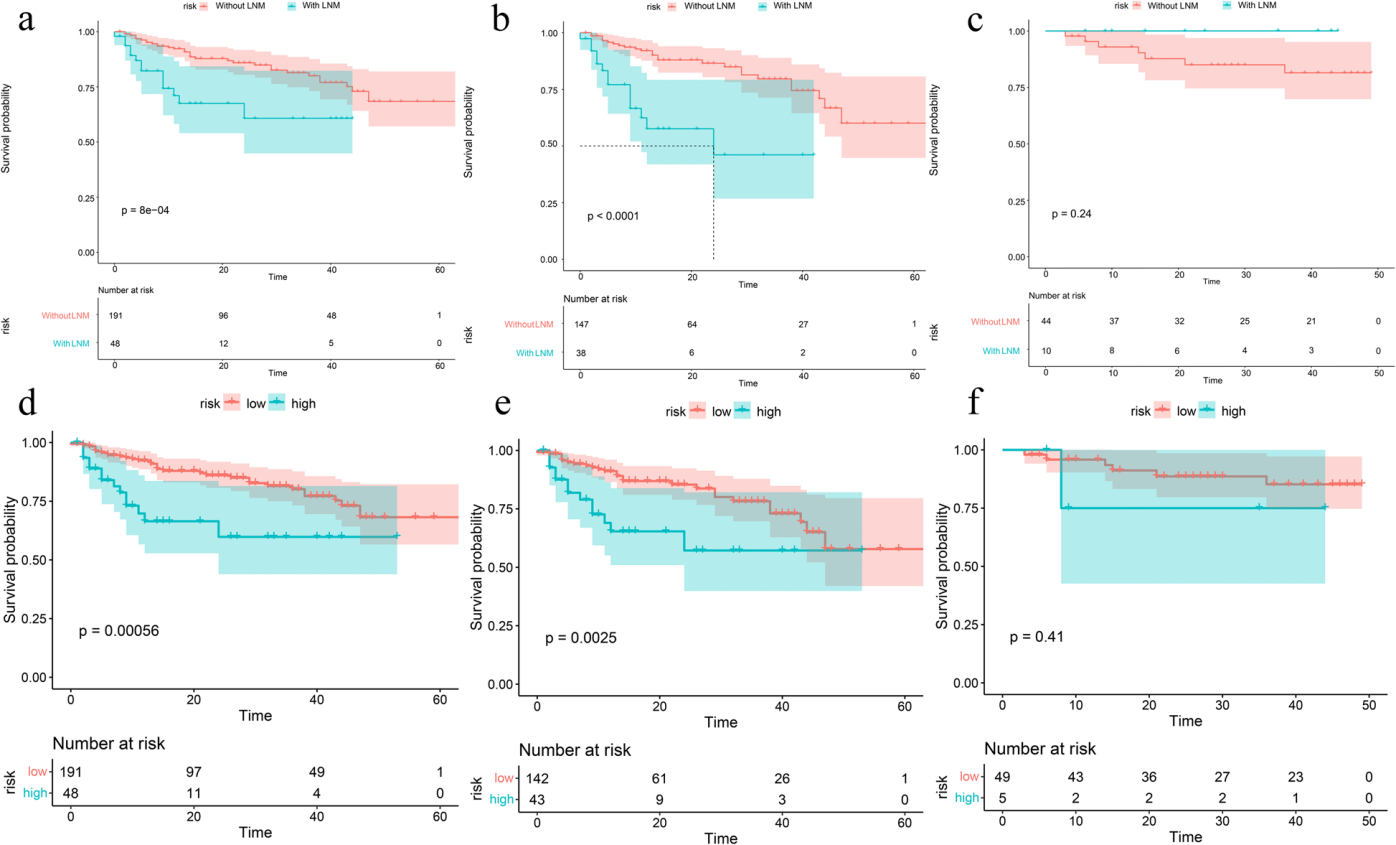
Survival analysis of the (**a**) pathologically confirmed LN status model (without LNM vs. with LNM) and (**d**) nomogram (low risk vs. high risk) in the total cohort. Survival analysis of the (**b**, **c**) pathologically confirmed LN status model and (**e**, **f**) nomogram in the training and external test sets, respectively. The nomogram showed significant differences for risk stratification in the total cohort and training set (log rank *p* < 0.05, respectively), but showed no statistical significance in the external test set (log rank *p* > 0.05, respectively)

Figure 4 shows the Kaplan–Meier survival curves based on the pathologically confirmed LN status model and combined model-predicted patient PFS. Over the total cohort and training set, combined model successfully stratifies the risk of patients (log rank *p* < 0.05, respectively).

## Discussion

LNM is an independent risk factor for death in BCa patients [17, 18]. The presence or absence of LNM significantly influences the selection of treatment strategies and prognosis [19]. In our study, we established and validated the combined model visualized as a nomogram that demonstrated excellent predictive performance. The combined model performs well in both the training (AUC: 0.980, 95% CI: 0.967–0.993) and external testing (AUC: 0.834, 95% CI: 0.659–1.000) sets, suggesting the potential to build a more generalized model for wider clinical use. For the external test set, the combined model had the highest predictive accuracy, indicating that it had less prediction error compared with other models. Precise prediction guides the selection of patients who require perioperative systemic chemotherapy integrated with extended LN dissection [20]. Furthermore, the combined model successfully stratifies patients into high-risk and low-risk, thereby suitably predicting PFS.

Radiology (e.g., CT and MRI) is now suggested for preoperative lymph nodal staging of BCa. Daneshmand et al. [21] found that the detection of metastatic malignant LN by CT or MRI relies on the LN’s size and has a sensitivity of only 31–45%, thus leading to the understaging of many patients. Therefore, judging the status of LN by the size is inaccurate because enlarged nodes may be the result of reactive hyperplasia, whereas small nodes may be positive for metastasis [22].

In previous studies, Tian et al. [23] screened low-dimensional clinical information such as age, grade, tumor size, and T-stage to develop a nomogram to predict LNM of BCa, achieving an AUC value of 0.704 for the test set. In our study, multivariate logistic analysis indicated that stalk presence and CT-reported LN status improve the construction of the clinical model. The AUC values of the clinical model were only 0.764 (95%CI: 0.697–0.831) for the training set and 0.624 (95CI: 0.402–0.846) for the external test set. This suggests that low-dimensional clinical information and visual features reflect only a small portion of the data in images and miss a significant quantity of detail with respect to lesion heterogeneity.

Radiomics has recently been proposed as a progressive computational methodology to extract quantitative descriptors from images of tumors [24]. Its application to vast amounts of medical images supports the diagnosis and treatment selection, and eliminates some of the shortcomings of traditional diagnostic approaches [25]. DL is one of the latest trends in artificial intelligence research [26], achieving revolutionary advances in computer vision and machine learning, and many notable breakthroughs in multiple fields. Li et al. [27] developed and validated a deep learning radiomics nomogram for CT images to predict LN status in gastric cancer, achieving promising discrimination performance on the test set (AUC: 0.821, 95% CI: 0.722–0.920). Wang et al [28]. built a nomogram based on DL and radiomics signatures to predict the axillary LNM in breast cancer, achieving high test set performance (AUC: 0.90, 95% CI:0.80–0.99). Thus, previous research demonstrates that combining conventional radiomics features with DL features is a promising approach for LNM assessment.

**Fig. 5:**
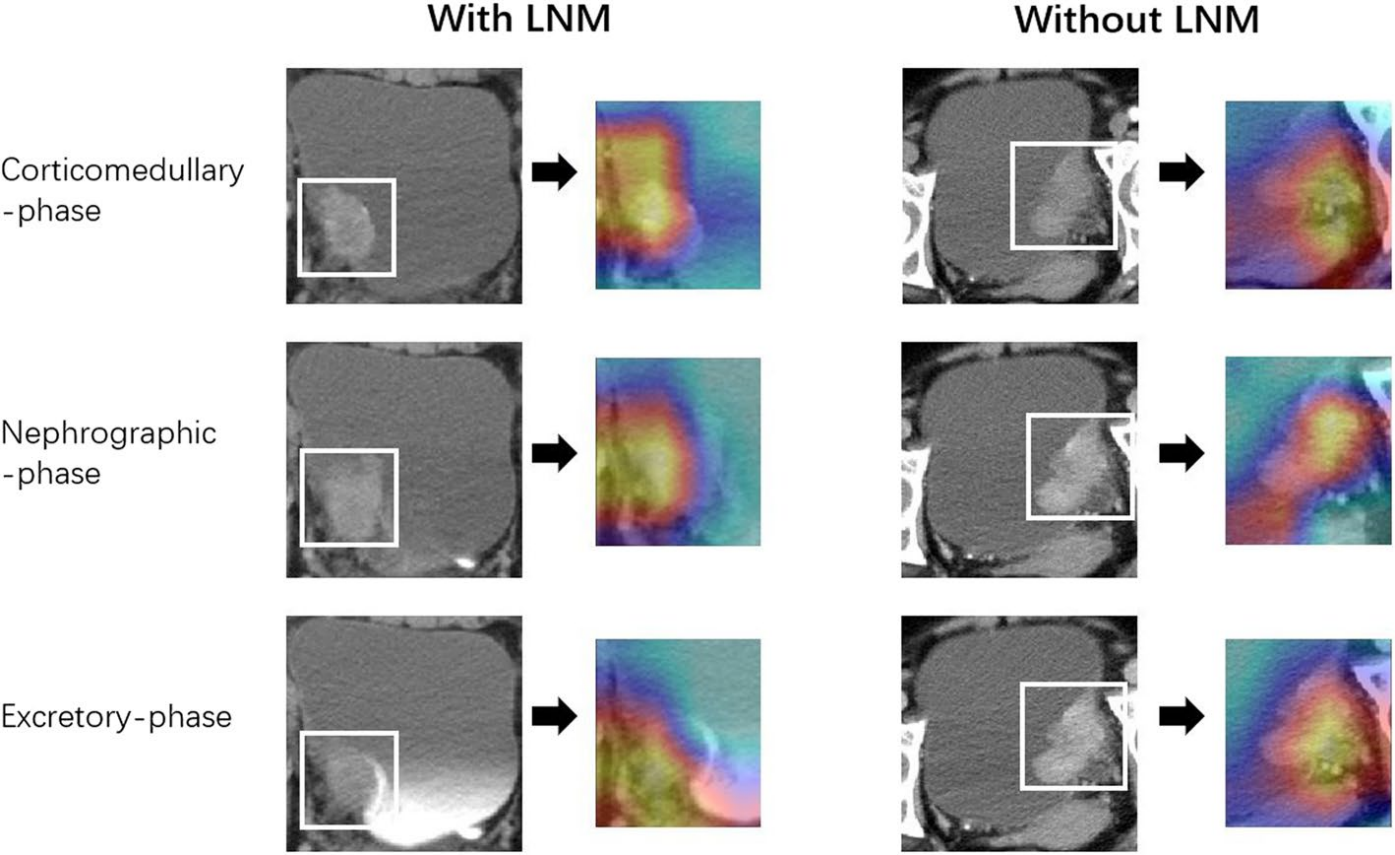
Activation maps of the deep convolutional neural networks for BCa LNM vs. non-LNM (reflecting the significant areas related to the risk of LNM) were obtained from three-phase CT. The yellow highlighted areas show strongly correspond with LNM predictions. The activation maps with LNM have a large range of yellow highlighted areas

In our study, the radiomics signature contained eight HCR features and four DL features, “A_wavelet.HHL_firstorder_Uniformity” demonstrated the greatest contribution among all the features. The wavelet features reflected the heterogeneity within tumors and better represented the image information [29]. Wavelet features allowed better outcome prediction and were a principal component in developing the radiomic signatures [30]. Convolutional neural networks also provided information related to LNM. As shown in Fig. 5, the activation maps highlighted the areas of the lesions associated with the LN status of BCa. We conclude that the yellow highlighted areas are strongly related to lesion metastasis in the activation maps.

Radiomics analysis, like other data-mining techniques, has dimensionality constraints [31]. Although the integration of feature screening methods and machine learning classifiers has reduced the dimensionality in big datasets, unsupervised clustering of imaging subtypes hinders accurate LN status prediction [32]. We combined the widely used mRMR and LASSO feature screening approaches with nine machine learning classifiers to determine the best radiomics machine learning signature to predict LN status. We determined that the LightGBM classifier retrained using SMOTE demonstrated excellent prediction ability. mRMR is an innovative feature screening method that uses more plausible coefficients and less redundancy to screen radiological features [33]. LASSO is a feature screening approach for avoiding over-fitting when building the model [34]. LightGBM is a classical machine learning algorithm for prediction [35]. Previous research showed that 25–30% of BCa patients who underwent radical cystectomy and pelvic lymph node dissection had LNM [36–38]. This is consistent with our data, where approximately one-fifth of all BCa patients showed LNM. Therefore, considering this unavoidable data imbalance, we used the SMOTE to retrain each machine-learning algorithm [39]. The AUCs of five of the nine machine learning signatures were improved by incorporating the SMOTE algorithm.

Patients with LNM had a poor prognosis. Many studies showed that the pathological status of pelvic LN is the independent predictor of death in BCa patients [40, 41]. Previous investigations showed that the prognosis of patients can be predicted by radiomics. Piotr et al. [42] discovered that radiomics features had prognostic value in predicting the overall survival of BCa patients. We tested combined model 's ability to forecast the prognosis of BCa patients. We found that combined model showed excellent risk stratification performance in the total cohort, suggesting that our model is promising for long-term management of BCa patients. However, the combined model showed no risk stratification difference for the external test set. A likely reason for this is that selection bias occurred in our external test set. In the training set, less than 8% (3/38) patients with pathologically confirmed LNM had PFS > 30 months, while in the external test set, 40% (4/10) patients with LNM had PFS > 30 months.

Our research still has several limitations. First, this was a retrospective study, and our results may be influenced by selection bias. Second, our study used manual segmentation to delineate the ROI, which may lead to deviations. Therefore, we intend to use automatic segmentation in future research [43]. Third, our data were gathered from two centers and varying CT scanners, so we used the combat compensation method to eliminate the negative influence of different protocols and CT scanners. Fourth, in order to standardize the development of predictive models, we referenced the Transparent Reporting of a multivariable prediction model for Individual Prognosis or Diagnosis (TRIPOD) [44] guideline and chose the type 3 to separate the training set and external test set. After that, we will try to group the patients randomly to explore whether the performance of the model is improved. Finally, we extracted radiomics features only from CT images, it’s uncertain whether multiparametric MRI is more helpful. In future studies, we will expand the sample size, in cooperation with international centers and add multidimensional data (e.g., MRI, ultrasound, genomics and pathology) to study the relationship between BCa lesions and LNM to improve the stability and generalization of the predictive models.

## Conclusion

Our proposed combined model using three-phase CT images is a non-invasive, readily available, and effective LNM prediction tool for BCa patients. We recommend its inclusion in BCa predictive models for improved monitoring and adjuvant clinical trial design to narrow the gap between radiology and precision healthcare.

### Supplementary Information

**Additional file 1:** **Table S1.** CT acquisition settings.

## Data Availability

The datasets used and/or analyzed during the current study are available from the corresponding author on reasonable request.

## Abbreviations

AdaBoost: Adaptive Boosting
AUC: Area under the receiver operating characteristic curve
BCa: Bladder cancer
CI: Confidence interval
DCA: Decision curve analysis
DL: Deep learning
HCR: Hand-crafted radiomics
ICCs: Intra- and inter- observer class correlation coefficients
KNN: K-Nearest Neighbor
LASSO: Least absolute shrinkage and selection operator
LightGBM: Light Gradient Boosting Machine
LN: Lymph nodes
LNM: Lymph node metastasis
LR: Logistic regression
MLP: Multilayer Perceptron
mRMR: Max-relevance and min-redundancy
PFS: Progression-free survival
ROI: Region of interest
SMOTE: Synthetic minority oversampling technique
SVM: Support vector machine
XGBoost: Extreme Gradient Boosting
