# Supplementary material for: Preoperative CT-based deep learning radiomics model to predict lymph node metastasis and patient prognosis in bladder cancer: a two-center study
Source: Insights Imaging. 2024 Jan 25;15:21. doi: 10.1186/s13244-023-01569-5 (PMC10811316; doi:10.1186/s13244-023-01569-5)
Supplement: Supplementary file 1 — Additional file 1: Table S1. CT acquisition settings. [file 13244_2023_1569_MOESM1_ESM.docx]

**Preoperative CT-based deep learning radiomics model to predict lymph node metastasis and patient prognosis in bladder cancer: a two-center study**

**ELECTRONIC SUPPLEMENTARY MATERIAL**

**Table S1** CT acquisition settings.

| **Parameters** | The Affiliated Hospital of Qingdao University | Shandong Provincial Hospital Affiliated to Shandong First Medical University |
| --- | --- | --- |
| **CT equipment** | BrightSpeed RT 16 Elite; Aquilion ONE 640, TOSHIBA; LightSpeed CT750 HD, GE Healthcare; SOMATOM CT, Siemens Healthcare; Somatom Sensation Cardiac 64, Siemens Healthcare; Brilliance iCT, Philips Healthcare; | Discovery 750, GE Healthcare; Somatom Definition Flash, Siemens Healthcare; Aquilion ONE, TOSHIBA |
| **Matrix size** | 512 × 512 | 512 × 512 |
| **Tube current** | automatic tube current modulation | automatic tube current modulation |
| **Pitch** | 0.8 | 0.8 |
| **Tube voltage** | 120 kV | 120 kV |
| **Slice thickness** | 5mm | 5mm |
| **Detector collimation** | 64×0.6mm  or 64×0.625mm | 64×0.6mm  or 64×0.625mm |
| **Contrast agent** | Ultravist 370  or Omnipaque 350 | Omnipaque 350 |
| **Injection rate** | 3.0ml/s | 3.0ml/s |
| **Contrast medium dose** | 80~90ml | 80~90ml |
